# Tracheostomy before and during COVID-19 pandemic

**DOI:** 10.2478/raon-2024-0034

**Published:** 2024-06-12

**Authors:** Sara Jensterle, Janez Benedik, Robert Sifrer

**Affiliations:** Department of Otorhinolaryngology and Cervicofacial Surgery, University Medical Centre Ljubljana, Ljubljana, Slovenia; Department of Anaesthesiology and Surgical Intensive Therapy, University Medical Centre Ljubljana, Ljubljana, Slovenia; Faculty of Medicine, University of Ljubljana, Ljubljana, Slovenia

**Keywords:** upper airway obstruction, emergent tracheostomy, urgent tracheostomy, anaesthesia, SARS-CoV-19, orotracheal intubation

## Abstract

**Background:**

The aim of the study was to provide insight into the influence of the COVID-19 on the frequency and characteristics of urgent and emergent tracheostomies (TS), comparing data collected both before and during the pandemic. Our two hypotheses were that *during COVID-19, more TS were performed in the emergent setting* and that *during COVID-19 more TS were performed under general anaesthesia*.

**Patients and methods:**

The research was retrospective. The study period included the two years before and after the COVID-19 outbreak in Slovenia. Forty-one patients in each period met the inclusion criteria. Their medical charts were reviewed. The anamnestic, clinical, surgical and anaesthesiological data were collected. The two groups of patients from corresponding time periods were statistically compared.

**Results:**

Predominantly men required the surgical resolution of acute upper airway obstruction (76% of patients). The causes for acute respiratory distress included head and neck cancer (62%), infections (20%), vocal cord paralysis (16%), and stenosis (2%). There were no statistically significant differences either in the (emergent/urgent) setting of TS or in the type of anaesthesia used. Both hypotheses were rejected. A statistically significant rise in use of the C-MAC laryngoscope during COVID-19 (from 3% to 15%) was reported.

**Conclusions:**

The outbreak of COVID-19 did not have a statistically significant effect on the frequency of performing emergent and urgent tracheostomies nor on the use of general or local anaesthesia. It did, however, require a change of intubation technique. Consequently, a significant rise in the use of the C-MAC laryngoscope was noted.

## Introduction

Acute respiratory distress (ARD) due to upper airway obstruction (UAO) is a life-threatening medical situation leading to, both, the imminent irreparable ischemic damage of the brain and/or cardiac arrest if not treated properly and promptly. These catastrophic events can occur in a matter of minutes. Thus, quick, determinate action is required in order to provide an alternative air conduit and ensure a clear, patent airway.^1,2,3^

After the identification of the site and degree of the UAO, which is the first step, further measures are taken to circumvent the obstructed airway. The first option is orotracheal intubation (OTI) most commonly performed by an anaesthesiologist under general anaesthesia. It is followed by an open tracheostomy (TS), which is usually performed by an otorhinolaryngologist or other appropriately, adequately trained surgical specialist.

Clinical conditions of ARD, where the patient can neither be intubated nor ventilated, are known as “cannot intubate-cannot ventilate” situations (CICV) and represent truly emergent clinical scenarios requiring a quick and effective surgical approach to the airway.^4^ The literature describes two surgical options for resolving ARD in the circumstances of CICV: the cricothyrotomy (CTT) and the tracheostomy (TS).

In December 2019, a new strand of Coronavirus, now named SARS-CoV-2 was discovered. Its outbreak negatively affected healthcare accessibility all over the world and, among other things, demanded the adaptation of surgical procedures to avoid viral transmission to healthcare providers. In regards to TS, the opening of the trachea and excision of tracheal window as the essential steps of the TS, might cause cough generating a large quantity of aerosol containing mucus, blood, and the virus. This would be directed towards the surgical and anaesthesiological teams, so the contamination of healthcare personnel with SARS-CoV-2 is highly probable during ordinary, i.e. not adapted, TS.^5^

There is a plethora of articles in the pertinent literature discussing the various surgical and anaesthesiological adaptations of the TS to the pandemic of COVID-19. Some of them were also proposed by our department.^4,6^

In this study, we chose to analyse the changing paradigm of ARD treated by TS as a result of the outbreak of COVID-19. The aim was to provide an in-depth comparison between the two eras (before the outbreak *vs*. during the COVID-19 pandemic) including the causes of ARD, the indications for emergent and urgent TS, the risk factors in the case of a difficult intubation, the surgical and anaesthesiological aspects of TS as well as the timing of the surgery. Our hypotheses firstly focused on the proposition that “*during the pandemic there were more TS performed in the emergent setting”*, and, secondly that “*during COVID-19 more TS were performed under general anaesthesia”*.

## Patients and methods

This retrospective study was conducted at the Department of Otorhinolaryngology and Cervicofacial Surgery at the University Medical Centre of Ljubljana, Slovenia. Medical charts, surgical and anaesthesiological reports from consecutive patients treated with TS for UAO associated with ARD during a four-year-long period, i.e., between 4^th^ of March 2018 and the 3^rd^ of March 2022 were reviewed. The data associated with the patient, ARD, the risk factors for difficult OTI, surgical establishment of alternative airway and anaesthesiological parameters were all systematically collected.

The patients were categorised into two groups, i.e., those managed during the COVID-19 pandemic (study group) and those treated before the outbreak of COVID-19 (control group). The dividing date was the 4^th^ of March 2020 as this was the day when the first case of COVID-19 was reported in Slovenia. Thus, the length of each period was exactly two years. The groups were statistically compared according to the above-mentioned parameters under evaluation.

From the point of a time-dependent aspect of TS, the TS were divided into emergent and urgent ones.^7^ For the purpose of our study, one of the following criteria had to be fulfilled for the definition of the emergent TS:

- The TS was performed on working days during regular hours immediately following the establishment of the UAO diagnosis. The on-going elective surgical program of the department was interrupted to carry out the TS.
- The TS was performed during “on duty” service.
- The TS was performed following the diagnostic direct laryngoscopy during which the imminent deterioration of the upper airway obstruction was recognised or suspected.
- The TS was performed in a CICV scenario.

On the other hand, if the dyspnoea was not severe enough to demand an emergent procedure, the TS was defined as urgent:

- The TS was not performed immediately after the establishment of UAO but scheduled for (at least) the following day.
- The TS did not fulfil the criteria for emergent TS

The elective TS performed, for example, in patients with curative or palliative treatment of head-and-neck cancer (HNC), with long-term OTI or with chronic aspiration were excluded from the study.

The statistical analyses were performed using the IBMI SPSS Statistics Version 25 (Chicago, IL). For comparative analyses, the Chi-Square test, Fisher’s exact test, t-test, and Mann-Whitney U test were used. All statistical tests were two-sided and *p*-values below 0.05 were considered statistically significant.

This study has been approved by the National Medical Ethics Committee of the Republic of Slovenia on 26^th^ of May 2022 under the number 0120-176/2022/3.

## Results

### All patients, both periods

A total of 82 patients were included into the study. The mean age of the patients was 69 years (range 28–97) and 62 (76%) of these were male.

From the surgical aspect, in the majority of the patients (72, 88%) the UAO was solved by means of a TS. The CTT as the first step in resolving upper airway obstruction was used in 6 cases (7%) and was transformed into TS immediately. Re-TS was performed in 4 cases (5%), meaning a patient already had a TS beforehand, and was successfully decannulated afterwards. From the aspect of emergency, the TS was considered emergent in 59 (72%) and urgent in 23 (28%) patients. In most of the cases, the surgery was performed by experienced otorhinolaryngologists, namely, in 68 patients (83%). In the remaining 14 cases (17%), the TS was performed by residents under the supervision of the experienced surgeon.

The UAO was most commonly caused by HNC (62%, 51 patients). The primary tumour sites included the larynx in 24 patients (47%), pharynx in 20 patients (39%), and other primary sites in 7 patients (14%). Other causes for the obstruction were infections including both mucosal upper airway infections and deep neck infections, as well as bilateral vocal cord paralysis and laryngotracheal stenosis. These results are detailed in Table 1. The UAO was caused by a single disease in 60 patients (73%), whereas in the remaining 22 patients (27%) multiple causes were registered. In these cases, the cause playing the most significant role was considered to be the main one.

**TABLE 1.** The main causes of upper airway obstruction

**Causes of upper airway obstruction**	**No. of patients**	**Ratio (%)**
All patients	82	100
Laryngeal cancer	24	29
Pharyngeal cancer	20	24
Cancer of other primary sites	7	9
Infections	16	20
Bilateral vocal cord paralysis	13	16
Laryngo-tracheal stenosis	2	2

Among the symptoms accompanying the dyspnoea, dysphagia was reported by 26 patients (32%), pain in 19 patients (23%), inspiratory stridor in 31 patients (38%), and biphasic stridor in a single patient (1%). The trismus was present in 6 cases (7%). Thirty-six patients were previously treated for various diseases of the head and neck: 14 patients (17%) by surgery, 11 patients (13%) received radiotherapy (RT) and 11 patients (13%) chemo-radiotherapy (CRT).

Taking into account the 51 patients with HNC as a cause of UAO requiring TS at the time of our study, 33 had de novo cancer while 18 had a recurrence or a new primary cancer. Specifically, 20% (10/51) had previously received RT and 16% (8/51) CRT.

Thirty-one patients had other causes of the UAO, namely infections, bilateral vocal cord paralysis and laryngotracheal stenosis. Thirteen percent (4/31) had a history of previous HNC and were treated by RT (one patient) and CRT (three patients).

Sixty-six (80%) patients had an available ASA score and were classified as ASA II (7%, 6/82), ASA III (53%, 44/82), and ASA IV (20%, 16/82).

Mallampati score was noted in 44 patients (54%, 44/82). Most of them (17%, 14/82) were ranked with the highest score 4, whilst score 3, 2 and 1 were attributed to 16% (13/82), 12% (10/82), and 9% (7/82) of patients, respectively.

Mouth opening was noted in 41 examinees. An adequate mouth opening was defined as an interincisor distance of more than 3 cm, as opposed to inadequate of less than 3 cm. Forty-one percent (34/82) of those were evaluated to have adequate mouth opening, whereas in 9% (7/82) it was inadequate.

Hyo-mental and thyro-mental distances were noted in 28 and 12 patients, respectively. Hyothyromental distance was the parameter coined by us for the purpose of the study and comprises the measurement of either of the two distances. It was obtained in 40 examinees. The distance was sufficient in 43% of the patients (35/82) and insufficient in 6% (5/82).

In 77 patients (94%), general anaesthesia was used, while in the remaining 5 patients (6%), the TS was performed under local anaesthesia.

The data, considering the type of the orotracheal tube, was able to be retrieved in 43 patients. Predominantly, a wire tube was used (39%, 32/82). A curved tube was used more seldomly (8 %, 7/82), while the straight tube was the least frequently used (5%, 4/82). The data concerning the manner of OTI in terms of the glottic exposure was reported in 67 cases. In 9% of the OTI (7/82), C-MAC video laryngoscope was used.

### A comparison of all patients regarding the period before and during COVID-19

Out of a total of 82 patients that were included in the study, 41 of them comprised the study group with the same number of patients included in the control group.

The parameters concerning the characteristics of the patients and prior treatments as well as the data associated with the actual disease causing UAO and its management from both a surgical and anaesthesiological perspective were statistically compared. The results of the statistical comparison between the study and control group are presented in Table 2.

**TABLE 2.** A comparison of the risk factors in all patients before and during the COVID-19 outbreak in Slovenia

**Risk factor**	**Overall**	**Before the outbreak of COVID-19**	**During the pandemic of COVID-19**	**p value**
All patients	82	41	41	
***Patients***				
Age (years) mean, range	66.8 (28–97)	64.8 (28–91)	68.8 (42–97)	0.172^a^
Male sex	62 (76%)	31 (76%)	31 (76%)	1.00^c^
Body weight (kg) mean, range	73.5 (35–143)	71.9 (35–143)	75.2 (43–110)	0.512 ^a^
Body height (cm) mean, range	172 (150–185)	172 (150–185)	173 (152–183)	0.933^a^
Body mass index (kg/m^2^), mean, range	25.4 (12.7–37.6)	24.2 (12.7–37.6)	27.6 (19,4–35,5)	0.178^a^
***The upper airway obstruction***				
Respiratory distress duration (days), median, range	6 (1–180)	10.5 (1–90)	3 (1–180)	0.373^b^
***Causes of acute airway obstruction***				0.826^c^
Laryngeal cancer	24 (29%)	12 (29%)	12 (29%)	
Pharyngeal cancer	20 (24%)	8 (20%)	12 (29%)	
Other cancers	7 (9%)	5 (12%)	2 (5%)	
Inflammation	16 (20%)	8 (20%)	8 (20%)	
Vocal cord paralysis	13 (16%)	7 (17%)	6 (15%)	
Laryngotracheal stenosis	2 (2%)	1 (2%)	1 (2%)	
Multiple causes	22 (27%)	11 (27%)	11 (27%)	1.000^c^
Respiration space (mm) mean, range	2.3 (1–5)	2.5 (1–4)	2.2 (1–5)	0.635^a^
***Symptoms***				
Dysphagia	26 (32%)	12 (29%)	14 (34%)	0.635^c^
Trismus	6 (7%)	5 (12%)	1 (2%)	0.101^d^
Pain	19 (23%)	8 (20%)	11 (27%)	0.432^c^
Stridor				0.504^c^
Inspiratory	31 (38%)	17 (42%)	14 (34%)	
Biphasic	1 (1%)	0	1 (2%)	
***Previous treatment***				
Surgery	14 (17%)	5 (12%)	9 (22%)	0.379^d^
RT	11 (13%)	5 (12%)	6 (15%)	1.000^d^
CRT	11 (13%)	5 (12%)	6 (15%)	1.000^d^
Surgery				
Re-TS	4 (5%)	0	4 (10%)	0.116^d^
Cricothyrotomy	6 (7%)	3 (7%)	3 (7%)	1.000^d^
Surgeon specialist	68 (83%)	34 (83%)	34 (83%)	1.000^c^
Time-dependent aspect of TS			0.806^c^	
Emergent	59 (72%)	29 (71%)	30 (73%)	
Urgent	23 (28%)	12 (29%)	11 (27%)	
Duration (hour) median, range	0.75 (0.25–2.50)	0.75 (0.25–2.5)	0.88 (0.25–2)	0.546^b^
***Anaesthesia***				1.000^d^
Local	5 (6%)	2 (5%)	3 (7%)	
General	77 (94%)	39 (95%)	38 (93%)	
Duration (hour) median, range	1.50 (0.75–3.25)	1.5 (0.75–3.25)	1.75 (1–3,25)	0.198^b^
ASA classification				0.952^c^
II	6 (7%)	3 (7%)	3 (7%)	
III	44 (53%)	23 (56%)	21 (51%)	
IV	16 (20%)	9 (22%)	7 (17%)	
Unknown	10 (20%)	6 (15%)	10 (25%)	
Mallampati classification				0.810 ^c^
I	7 (9%)	5 (12%)	2 (5%)	
II	10 (12%)	6 (15%)	4 (10%)	
III	13 (16%)	7 (17%)	6 (14%)	
IV	14 (17%)	7 (17%)	7 (17%)	
Unknown	38 (46%)	16 (39%)	22 (54%)	
Mouth opening				0.207^d^
Inadequate	7 (9%)	6 (15%)	1 (2%)	
Adequate	34 (41%)	18 (44%)	16 (39%)	
Unknown	41 (50%)	17 (41%)	24 (59%)	
Hyo/thyromental distance				1.000^c^
Insufficient	5 (6%)	3 (7%)	2 (5%)	
Sufficient	35 (43%)	21 (51%)	14 (34%)	
Unknown	42 (51%)	17 (42%)	25 (61%)	
Endotracheal tube type				0.072^c^
Wire	32 (39%)	18 (44%)	14 (34%)	
Straight	4 (5%)	4 (10%)	0	
Curved	7 (8%)	2 (5%)	5 (12%)	
Unknown	39 (48%)	17 (41%)	22 (54%)	
Orotracheal intubation				0.043^d^^*^
C-MAC laringoscope	7 (9%)	1 (3%)	6 (15%)	
Laryngoscope	60 (73%)	35 (85%)	25 (61%)	
Unknown	15 (18%)	5 (12%)	10 (24%)	

ASA = American Society of Anaesthesiologists; COVID-19 = Coronavirus infectious disease 19; CRT = chemo-radiotherapy; RT = radiotherapy; TS = tracheostomy

a = T test,

b = Mann-Whitney U-test,

c = hi-square test,

d = Fisher exact test

Regarding the main questions of the study, there were no differences between the study and control group in our research. Namely, before the outbreak of COVID-19, TS was performed in the emergent setting in 71% (29/41) of patients, whereas during the COVID-19 pandemic, that number was 73% (30/41). Thus, there is no statistically significant difference demonstrated (p = 0.806). Furthermore, there was no statistically significant difference in relevance to the general anaesthesia (p = 1.000), either, as before the era of COVID-19, TS was performed under general anaesthesia in 95% (39/41) and during the pandemic in 93% (38/41) of patients.

Nevertheless, Table 2 gives us insight into some differences between the two groups, with the most prominent ones implicated through endotracheal intubation. A trend of less frequent use of the straight orotracheal tube during the pandemic (0%, 0/41) in comparison to the time prior to the COVID-19 (10%, 4/41) outbreak has been noted. There is an increase in the use of the curved tube from 5% (2/41) to 12% (5/41), the difference is, however, not statistically significant (p = 0.072). The change in implementation of the C-MAC video-laryngoscope proved to be statistically significant (p = 0.043). Before COVID-19, it was used in 3% (1/41) of cases and in 15% (6/41) of cases during COVID-19.

### A comparison of patients with HNC regarding the period before and during COVID-19

Since some of the risk factors are specific for patients with HNC (such as prior RT and CRT) and not for patients with other diseases, another comparison was made. This included 51 patients with HNC, 26 of whom were in the study group and 25 in the control group. The results are depicted below in Table 3.

**TABLE 3.** A comparison of the risk factors in patients with HNC before and during the COVID-19 outbreak in Slovenia

**Risk Factor**	**Overall**	**Before the Outbreak of COVID-19**	**During the Pandemic of COVID-19**	**p value**
All Patients	51	25	26	
***Patients***				
Age (years) mean, range	66.2 (28–88)	64.6 (28–88)	67.8 (42–87)	0.374 ^a^
Male sex	44 (86%)	22 (88%)	22 (85%)	1.000^d^
Primary site				0.365 ^c^
Laryngeal cancer	24 (47%)	12 (48%)	12 (46%)	
Pharyngeal cancer	20 (39%)	8 (32%)	12 (46%)	
Other cancers	7 (14%)	5 (20%)	2 (8%)	
***Invasion of subsites***				
Glottis	21 (42%)	13 (52%)	8 (31%)	0.124^c^
Supraglottis	32 (63%)	16 (64%)	16 (62%)	0.856^c^
Subglottis	11 (22%)	6 (24%)	5 (19%)	0.743^d^
Trachea	2 (4%)	1 (4%)	1 (4%)	1.000^d^
Hypopharynx	13 (26%)	3 (12%)	10 (39%)	0.052^d^
Oropharynx	16 (31%)	10 (40%)	6 (23%)	0.193^c^
Oral cavity	8 (16%)	4 (16%)	4 (15%)	1.000^d^
Respiration Space (mm), mean, range	2.4 ± 1.5 (1–5)	3 ± 1.7 (1–4)	2.2 ± 1.5 (1–5)	0.714 ^b^
***Previous treatment***				
Surgery	8 (16%)	3 (12%)	5 (19%)	0.703^d^
RT	10 (20%)	4 (16%)	6 (23%)	0.726^d^
CRT	8 (16%)	4 (16%)	4 (15%)	1.000^d^
***Surgery***				
Time-dependent aspect of TS				0.806 ^c^
Emergent	34 (67%)	16 (64%)	18 (69%)	
Urgent	17 (33%)	9 (36%)	8 (31%)	
Orotracheal intubation				0.047^d^*
C-MAC	7 (14%)	1 (4%)	6 (23%)	
Laryngoscope	38 (74%)	22 (88%)	16 (62%)	
Unknown	6 (12%)	2 (8%)	4 (15%)	

COVID-19 = coronavirus infectious disease 19; CRT = chemo-radiotherapy); RT = radiotherapy, TS = tracheostomy

a = *T test,*

b = *Mann-Whitney U-test,*

c = *hi-square test,*

d = *Fisher exact test*

There were no differences in the primary tumour sites between the two periods. Nevertheless, we observed a trend of higher incidence in the invasion of hypopharynx in the study group as opposed to the control group (39% *vs*. 12%, p = 0.052).

There was also a significant rise in the use of C-MAC during COVID-19, from 4% to 23% (p = 0.047).

## Discussion

### The first hypothesis

Throughout the study period, the TS due to ARD caused by UAO was performed in 72% as an emergency surgical procedure. The rates of emergent TS before and during the COVID-19 pandemic were 71% and 73%, respectively. This difference did not attain statistical significance. Therefore, our first hypothesis stating that “*during the pandemic there were more TS performed in the emergent setting”* was rejected.

During the epidemic, access to sport and outdoor activities was severely limited. As physical activity diminished, the average body mass index rose from 24,2 to 27,6 kg/m^2^, so not significantly. Similarly, patients’ access to their general practitioners was also limited so patients received no regular medical attention. The cancers, inflammations and other medical conditions progressed unimpededly leading to higher stages of diseases and more clinical problems when patients finally found their way to their doctors. In this way, the suboptimal accessibility of general practitioners could explain the (not significantly) decreased width of the airway – from 2.5 to 2.2 mm at the narrowest point of the airway. An elevated BMI and decreased width of airways led us to expect a higher rate of difficult intubations and emergent TS in COVID-19 on account of the urgent ones. However, this was not the case, as the difference did not emerge as significant.

For the same reasons, more patients with CICV situations were expected. The literature offers two options to treat ARD in a CICV scenario: CTT and TS.^8^ In emergent situations, otorhinolaryngologists, as a general rule, prefer TS, which is supported by our results – 93% of patients received TS, whereas only 7% received CTT, which was then immediately converted into TS. The decision for (a more complicated, longer and riskier) TS as opposed to a (technically less demanding and speedier) CTT in the emergent setting is surprising. Moreover, The Advanced Trauma Life Support (ATLS) guidelines recommend CTT in an emergency CICV situation.^9^ Nevertheless, the otorhinolaryngologists are trained in emergent surgical airway management very early on in their careers, so the educational goals in the residency programmes prepare them to perform TS within a few minutes.^4,10^ This actually means that the otorhinolaryngologists are more experienced in performing TS than CTT explaining the low rate of CTT on account of TS in an emergency setting. However, for all other specialists who may not have TS in their residency programme, the CTT is suggested followed by the referral of the patient to the closest otorhinolaryngological unit.

There was an equal rate of CTT during both periods – 7%. A higher rate was expected during COVID-19 as the Slovenian national guidelines for emergent TS during the era of COVID-19 advised CTT as the first step in emergent TS.^4^ However, the guidelines target a CICV situation in COVID-19 patients or those with an unknown COVID-19 status. Since the incidence of true CICV situations is only 0.4%^11^ the amount of CTT performed before and during COVID-19, remained the same.

### The second hypothesis

The TS were performed over the entire four-year-long period, mainly under general anaesthesia – in 94% of cases. Again, there are no statistically significant differences demonstrated in the periods before or during the pandemic, where general anaesthesia was used in 93% and 95% of cases, respectively. Therefore, our second hypothesis claiming that “*during COVID-19 more TS were performed in the general anaesthesia”* was also rejected.

Irrespective to the studied periods, we noticed a significantly higher percentage of TS under general anaesthesia as compared to reports from the literature.^12,13^ At first, TS under local anaesthesia is much more unpleasant and uncomfortable for the patient as well as for the surgeon. Furthermore, the dyspnoeic, restless and hypoxic patients frequently do not cooperate with the surgical team and impede the course of the surgery. In addition, the opening of the trachea results in the generation of aerosol potentially transmitting the SARS-CoV-2 and/or other diseases to the health personnel.^4,5^ To conclude, TS under local anaesthesia is associated with a great many issues and is, therefore, avoided. This applies to all patients and does not depend on the presence of the COVID-19.

Secondly, with the advent of sophisticated equipment such as C-MAC, the bonfils endoscope and procedures such as transnasal awake fiberoptic intubation^11,14^, the endotracheal intubation almost always succeeds. This would explain the high rate of implementation of general anaesthesia in TS. Therefore, TS under local anaesthesia is reserved only for occasional occurrences of CICV.

Thirdly, as TS under general anaesthesia generates less aerosol due to the surgical technique adjustment, we expected fewer TS under local anaesthesia during COVID-19. We attribute the lack of the expected difference to the fact that there were not many CICV occurrences in the studied period, as they are rare, per se.^11^ According to the Slovenian guidelines in the case of an occurrence of CICV in COVID-19, CTT under general anaesthesia is proposed as one of the essential steps of the emergent TS in patients with COVID-19.^4,15^ CICV incidence was not determined in our study, however, with an incidence of 0.4% from the literature^11^, we expected only occasional CICV cases.^11,16,17^

### Other comparisons

In the majority of OTI, a wire tube was used (32 patients, 39%), which is also recommended in surgical procedures and difficult intubations.^18^ There was a trend demonstrating the less frequent use of the straight tube (10% *vs*. 0) and more frequent use of the curved endotracheal tubes (5% *vs*. 12%) during the pandemic. The preference for curved tubes during COVID-19 could be explained by its convenient use in combination with C-MAC, especially in the case of a difficult intubation. Curved tubes reduce the risk of obstruction due to folding, in comparison to a straight tube. The diversity of tube types in the study could also be attributed to various anaesthesiologists being involved in emergent TS and therefore a deviation from regular intubation protocol.

The increased use of C-MAC during COVID-19 proved to be statistically significant, as it rose from 3% to 15%. What is more, it was also considered statistically significant during a separate analysis of patients with HNC (4% *vs*. 23%). This can be attributed to international guidelines, which recommend C-MAC video-laryngoscopes in patients with confirmed or suspected COVID-19 since it enables a further and, consequently, safer distance between the anaesthesiologist’s face and patient’s mouth, therefore minimising the anesthesiologist’s exposure to the contaminated aerosol.^19^ Video laryngoscopes enable the anaesthesiologist to execute the intubation and observe the insertion of the tip of the tube on a monitor rather than looking directly into the patient’s airway. An additional benefit to this method is the shorter intubation time required.^18^

An interesting trend was also noted from the perspective of tumour subsites invasion. After the separate analysis of patients with HNC, we noted an increase in HNCs invading the hypopharynx. This rose from 12% in the control group to 39% in the study group. This trend could be attributed to the fact that patients, due to COVID-19, sought medical help later, with more widely spread cancer.

The appearance of the SARS-CoV-2 had a significant impact on the management of UAO, both in Slovenia and around the world. Although COVID-19 changed the surgical and anaesthesiological perspectives of the management of ARD in patients with UAO, emergent TS remains one of the most important and time-honoured solutions. The study compared the time period before the onset of COVID-19 to that during the epidemics. Eighty-two patients were included in the study with 41 in each observed period.

The elderly male patients were (not significantly) more often affected by ARD caused by UAO and required intervention more often than younger, female counterparts. The UAO was most often caused by HNC (62%), followed by patients with inflammatory diseases (20%) and recurrent laryngeal nerve palsy (16%). Among HNC, the laryngeal (47%) and pharyngeal cancer (39%) predominated.

## Conclusions

In terms of TS, the comparison between the two eras (before the outbreak *vs*. during the COVID-19 pandemic) revealed no significant differences neither in the proportions of emergent and urgent TS nor in use of general or local anaesthesia. However, the C-MAC video laryngoscope was (statistically significantly) more often used during COVID-19 (from 3% to 15%) which goes hand in hand with the international anaesthesiological guidelines.
